# Genome-wide binding analysis of the transcriptional regulator TrmBL1 in *Pyrococcus furiosus*

**DOI:** 10.1186/s12864-015-2360-0

**Published:** 2016-01-08

**Authors:** Robert Reichelt, Antonia Gindner, Michael Thomm, Winfried Hausner

**Affiliations:** Lehrstuhl für Mikrobiologie und Archaeenzentrum, Universität Regensburg, Universitätsstrasse 31, Regensburg, D-93053 Germany

**Keywords:** Archaea, Transcription factor, Transcription regulation, TrmB, TrmBL1, TGM, ChIP-seq

## Abstract

**Background:**

Several *in vitro* studies document the function of the transcriptional regulator TrmBL1 of *Pyrococcus furiosus*. These data indicate that the protein can act as repressor or activator and is mainly involved in transcriptional control of sugar uptake and in the switch between glycolysis and gluconeogenesis. The aim of this study was to complement the *in vitro* data with an *in vivo* analysis using ChIP-seq to explore the genome-wide binding profile of TrmBL1 under glycolytic and gluconeogenic growth conditions.

**Results:**

The ChIP-seq analysis revealed under gluconeogenic growth conditions 28 TrmBL1 binding sites where the TGM is located upstream of coding regions and no binding sites under glycolytic conditions. The experimental confirmation of the binding sites using qPCR, EMSA, DNase I footprinting and *in vitro* transcription experiments validated the *in vivo* identified TrmBL1 binding sites. Furthermore, this study provides evidence that TrmBL1 is also involved in transcriptional regulation of additional cellular processes e.g. amino acid metabolism, transcriptional control or metabolic pathways. In the initial setup we were interested to include the binding analysis of TrmB, an additional member of the TrmB family, but western blot experiments and the ChIP-seq data indicated that the corresponding gene is deleted in our *Pyrococcus* strain. A detailed analysis of a new type strain demonstrated that a 16 kb fragment containing the *trmb* gene is almost completely deleted after the first re-cultivation.

**Conclusions:**

The identified binding sites in the *P. furiosus* genome classified TrmBL1 as a more global regulator as hitherto known. Furthermore, the high resolution of the mapped binding positions enabled reliable predictions, if TrmBL1 activates (binding site upstream of the promoter) or represses transcription (binding site downstream) of the corresponding genes.

**Electronic supplementary material:**

The online version of this article (doi:10.1186/s12864-015-2360-0) contains supplementary material, which is available to authorized users.

## Background

Basal transcription in archaea relies on a eukaryotes-like transcription machinery and promoter elements, whereas, transcriptional regulation is based on mainly bacteria-like transcriptional regulators [1–3]. These regulators can act as repressors [4,5], activators [6–8] or both [9–11]. In the genome of the hyperthermophilic euryarchaeon *Pyrococcus furiosus* a total number of 85 putative transcription factors (TFs) can be found, which represent about 4 % of all open reading frames (ORFs) [12]. 13 of these regulators belong to the TF family of TrmB (transcriptional regulator of mal operon) proteins, which is mainly distributed within the euryarchaeota, but can be found across all archaeal divisions [12–14].

Two of them, TrmB and TrmBL1 (TrmB-like protein 1), play a crucial role in transcriptional control of genes involved in sugar transport and metabolism in *P. furiosus* [10,14–16]. TrmB mainly serves as transcriptional repressor of the operon encoding an ABC transporter specific for trehalose and maltose (TM-system) [17–19]. The TrmB binding site in the TM system overlaps TFB-recognition element (BRE) as well as TATA-box and repression is mediated by impairing TATA-binding protein (TBP) and transcription factor B (TFB) binding through steric hindrance [18]. In contrast, TrmBL1 functions as a global regulator, which can act both as repressor and activator [10,11]. The *Thermococcales*_Glycolytic_Motif (TGM) with the consensus sequence TATCAC-N5-GTGATA serves as palindromic DNA recognition element for TrmBL1 binding *in vitro* and *in vivo* [10,11,20]. Targets of TrmBL1 mediated regulation are genes encoding enzymes mainly involved in sugar uptake, glycolysis, and gluconeogenesis. The dual functionality of TrmBL1 relies on binding upstream or downstream of the promoter elements [10,11]. TrmBL1 binding downstream of the TATA-box inhibits RNA polymerase (RNAP) recruitment, whereas upstream binding activates transcription.

A detailed *in vitro* analysis of TrmB and TrmBL1 revealed crossregulation of both factors on some promoters, e.g. the TM and maltodextrin-specific-ABC-transporter (MD) system [10]. Thus, we chose the chromatin immunoprecipitation (ChIP) approach to decipher the specific genomic binding sites of TrmB and TrmBL1 *in vivo*. Currently, only a few ChIP studies exploring archaeal species are available, mainly for the halophilic strain *Halobacterium salinarum*-NRC1 and the hyperthermophilic crenarchaea *Sulfolobus solfataricus* and *Sulfolobus acidocaldarius* [21–26]. Most of these groups combined ChIP with whole genome microarray analysis (ChIP-chip) for the analysis of genome-wide protein occupancies. Instead of using ChIP-chip, coupling of ChIP with high-througput sequencing (ChIP-seq) became a widely used approach for quantitative mapping of protein-DNA binding events in a genome-wide manner in eukaryotic and bacterial systems [27,28]. Recently, a workflow for genome-wide mapping of archaeal transcription factors ChIP-seq was reported [29].

The aim of this study was to dissect the specific role of TrmB and TrmBL1 as transcriptional regulators of genes encoding enzymes involved in sugar uptake, glycolysis and gluconeogenesis in a genome-wide manner *in vivo*. For this purpose a previously described ChIP protocol of our group for the hyperthermophilic archaeon *P. furiosus* was successfully improved for a ChIP-seq approach [30]. The identified binding sites in the *P. furiosus* genome under steady state glycolytic or gluconeogenic growth conditions exposed the function of TrmBL1 as global regulator for sugar transport and metabolism and revealed novel and unexpected genes which are in addition under the transcriptional control of TrmBL1.

## Methods

### Strain and media

*P. furiosus* type strain DSM3638 was obtained from the *Deutsche Sammlung von Mikroorganismen und Zellkulturen* (DSMZ) recently and after growth in SME complex media the strain was prepared for long time storage. Cells were grown under anaerobic conditions in nutrient rich medium based on SME [31] and supplemented with different organic substrates. Complex SME media contained 0.1 % starch, 0.1 % peptone and 0.1 % yeast extract. SME starch media contained 0.1 % starch and 0.025 % yeast extract. For SME pyruvate medium starch was replaced by 40 mM pyruvate and SME maltose contained 5 % maltose instead of starch. After inoculation with *P. furiosus* cells (1:100 dilution) cultivation was done at 95 °C overnight or until the appropriate cell density was reached.

### Plasmids and primers

All used plasmids and primers are shown in the supplement (Additional file 1).

### Quantitative real-time PCR (qPCR)

qPCR pimerpairs were designed using the Primer3 software package and quality assessed [32–34]. qPCR reactions were assembled as duplicates or triplicates in a total volume of 10 μl using the SensiMix™ SYBR® No-ROX Kit (Bioline, Luckenwalde, Germany). Primers were added to a final concentration of 0.3 μM and the total volume of the DNA samples in each reaction was 4 μl. No-template-control using EB buffer (QIAquick PCR purification kit, Qiagen, Hilden, Germany) was included for every primer pair. qPCR reactions were run on a Rotorgene6000 platform (Corbett, Sidney, Australia) using a three step protocol with an annealing temperature of 58 °C for every primer pair. Data analysis was done using the corresponding Rotorgene software package (Qiagen, Hilden, Germany). Only qPCR reactions with an efficiency of 0.8 to 1.2 for the corresponding primer pair were considered as determined by dilution serious. The specificity of the PCR products was verified by melt curve analysis. Moreover, duplicate or triplicate reactions with a standard deviation (SD) > 0.5 quantification cycle (Cq) values were excluded from analysis.

### Copy number analysis

Genomic DNA was extracted from the following *P. furiosus* cell cultures: recultivation culture and from three biological replicates after two or five transfers in minimal SME starch, pyruvate or maltose [35]. Copy number of the genes PF1753 and PF1743 was determined by qPCR and the relative quantification method [36] using the gene PF1784 as calibrator and the recultivation culture as reference for the cells grown under the three different conditions. Sequences of corresponding primer pairs for PF1743, PF1753 and PF1784 are shown in Additional file 1. The results are represented as mean of the three biological replicates including SD.

### Southern blot analysis

Southern blot analysis was done as described previously [35]. Total genomic DNA was digested with BamHI and SmaI. For probe labeling DNA fragments were amplified by PCR. One probe specifically detects a 7 kb fragment harbouring the gene PF1743 and the other a 3 kb fragment with the gene PF1753. Molar ratios of both probes were adjusted to achieve comparable signal intensities.

### Antibody production and purification

Polyclonal rabbit antibodies were produced by Davids Biotechnology (Regensburg, Germany) using recombinantly expressed and purified TrmB and TrmBL1 proteins [10]. The IgG fraction of the polyclonal antibodies was purified using an immobilized Protein G column (GE Healthcare, Uppsala, Sweden) according to the manufactures instructions. Antibody containing fractions were pooled and dialyzed in PBS overnight. The protein concentration was determined by Bradford assay.

### Western blot analysis

The recombinantly expressed and purified proteins TrmB, TrmBL1 and TrmBL2 were obtained as previously described [10,17,37]. Cell extracts from *P. furiosus* were prepared from 20 ml cell cultures with a cell density of approximately 1x10^8^ cells per ml. After harvesting cells were resuspended in PBS supplemented with protease inhibitor mix (cOmplete Ultra Tablets, Roche Applied Science, Mannhein, Germany) and treated with glass beads using a FastPrep24 (M.P.Biomedicals, Irvine, USA) for cell lysis. After removal of cell debris by centrifugation the protein concentrations of the supernatants were determined by Bradford assay. Western blot experiments were done as described previously [35].

### Chromatin Immunoprecipitation

Formaldehyde crosslinking was done according to Liu et al. [30]. After recultivation in SME complex medium, *P. furiosus* cells were transferred to SME minimal medium supplemented with either starch (glycolytic inoculation culture 1) or sodium pyruvate (gluconeogenic inoculation culture 1) for adaptation to glycolytic or gluconeogenic growth conditions. Moreover, *P. furiosus* cells grown under gluconeogenic conditions were transferred additional three times in SME minimal medium supplemented with pyruvate (gluconeogenic inoculation culture 2). These three cell cultures were used for cultivation of *P. furiosus* in 15 L bio-fermenters containing the appropriate medium (samples: glycolytic culture 1 (starch 1) and gluconeogenic cultures 1 and 2 (pyruvate 1 and 2)). After the cells reached a cell density of 1 to 1.5x10^8^ cells/ml (middle to late exponential phase) fixation was done directly in the bio-fermenter at 95 °C with a final concentration of 0.1 % (v/v) formaldehyde. After 20s the crosslinking reaction was stopped by adding glycine to a final concentration of 15 mM and the bio-fermenter was straight away cooled down to 20 °C for harvesting the crosslinked *P. furiosus* cells [30].

Formaldehyde-treated cells were disrupted by sonication using the Branson Sonifier (Branson, Danbury, USA) until an average fragment length of 250 bp to 500 bp was obtained (Additional file 2). Insoluble particles were removed by centrifugation. After freezing with liquid nitrogen the cell extracts were stored at −80 °C. For determination of DNA concentration and fragment length 1 volume of cell extract was mixed with 4 volumes ChIP elution buffer (10 mM Tris pH 8.0, 1 % (w/v) SDS, 0.1 mM EGTA) and incubated overnight at 65 °C. After RNase treatment DNA was purified via the QIAquick PCR Purification Kit (Qiagen, Hilden, Germany) and concentration was measured using the NanoDrop (Peqlab, Erlangen, Germany).

For immunoprecipitation (IP) 5 μg of purified polyclonal antibodies raised against TrmBL1 or Phr was coupled to 50 μl Dynabeads Protein G for immunoprecipitation (Thermo Fisher Scientific, Waltham, USA) according to the manufactures instructions. Antibody coupled magnetic beads were resuspended in 500 μl *P. furiosus* cell extracts adjusted to a total DNA amount of 15 μg in PBS and incubated overnight at 4 °C. Immunoprecipitated complexes were washed in total five times with 500 μl of the following washing buffers: 2x low salt buffer, 1x high salt buffer, 1x LiCl detergent and 1x TE [38]. Elution from the beads was done in 100 μl ChIP elution buffer at 65 °C for 10 min. A second elution step was done without heating using 150 μl TE buffer supplemented with 0.67 % (v/v) SDS and both eluates were combined. For the input sample 200 μl TE supplemented with 1 % (v/v) SDS was added to 50 μl not immunoprecipitated *P. furiosus* cell extract (1,5 μg total DNA, 10 % of the IP; starch 1 input, pyruvate 1 input and pyruvate 2 input). Eluted complexes and input samples were incubated overnight at 65 °C for reversal of the crosslink. After treatment with RNase A and Proteinase K ChIP enriched and input DNA was purified via the QIAquick PCR purification kit (Qiagen, Hilden, Germany) and stored at −20 °C. For each culture (glycolytic culture/starch 1 and gluconeogenic culture/pyruvate 1 and 2) the immunoprecipitation step was repeated once (starch 1 IP1 and 2; pyruvate 1 IP1 and 2 and pyruvate 2 IP1 and 2).

### Library preparation and sequencing

Library preparations were done according to the NEBNext ®-ChIP-Seq library prep reagent set for Illumina protocol (New England Biolabs, Ipswich, USA). For multiplex sample preparation the NEBNext® Multiplex oligos (primer set 1 and 2) (New England Biolabs, Ipswich, USA) were used and libraries were PCR amplified by the NEBNext®High Fidelity Master Mix (New England Biolabs, Ipswich, USA). Libraries were pooled in equimolar ratios and sequenced using the Illumina HiSeq 2000 platform (read length = 50 b) (Illumina, SanDiego, USA). For further analysis only the demultiplexed and quality filtered reads (Eland, Illumina, SanDiego, USA) were used.

### Data processing and peak calling

Reads were uploaded to the galaxy server platform [39] and mapped to the *P. furiosus* DSM3638 genome using Bowtie2 with default settings [40]. Aligned and unaligned reads were written to different files. Peak calling was performed using Model-based analysis for ChIP-seq (MACS2 (2.1.0)) [41] using default settings with following exceptions: Effective genome size = 1.90E + 6; band width = 300; model fold = [1,100]; *Q*-value cutoff = 1.00E-5 and the maximum duplicate tags at the same position were adjusted to the minimal value sufficient for building the paired-peak model. Following sample combinations were analysed: starch 1 IP1 versus starch 1 input, starch 1 IP2 versus starch 1 input, pyruvate 1 IP1 versus pyruvate 1 input, pyruvate 1 IP2 versus pyruvate 1 input, pyruvate 2 IP1 versus pyruvate 2 input and pyruvate 2 IP2 versus pyruvate 2 input. Using these settings no significant peaks were detected in the samples starch 1 IP1 and 2. Moreover, from the peaks called in the samples pyruvate 1 IP1 and 2 and pyruvate 2 IP1 and 2 only those were considered for further analyses which were present in at least three from the four samples (Additional file 3).

### ChIP qPCR

The ChIP-enriched DNA was also measured by qPCR using the % input method [42]. qPCR reactions were performed as described above. The corresponding primer pairs are shown in the Additional file 1. Considering that only 10 % of the IP sample volume was used for the input sample % input was calculated by the formula: 100 * 2 ^ (Cq (adjusted Input) - Cq (IP)). % input values are shown as mean of at least three technical replicates of IP with SD.

### De-novo motif discovery and promoter scan

The consensus TF binding site for each peak region was calculated as midpoint of the sites detected by MACS2 in the different samples. The DNA sequences surrounding these midpoints in the range of 350 bp downstream and upstream were used for De-novo motif discovery by MEME [43,44] using default settings with following exceptions: number of motifs = 5; minimum width = 10 and maximum width = 25). The identified motif displaying highest significance was further analysed by additional tools. Analysis of motif enrichment was performed by AME (default settings) [45]. Additionally, motif occurrences in the *P. furiosus* DSM3638 genome were searched by FIMO [46] using a *P*-value cut-off < 1.0E-5. Analysis of centrally enrichment was done by Centrimo (default settings) [47].

Scanning for promoter occurrences in the detected ChIP-enriched was performed by FIMO [46] search using a *P*-value cut-off < 1.0E-3. The 14 bp consensus sequence of the BRE- and TATA-box (based on 27 *P. furiosus* promoter sequences) was loaded as position-frequency matrix reported by vandeWerken et al. [20]. The scan was done in the 34 TGM containing peak regions surrounding the TF binding site midpoints in the range of 1000 bp upstream and downstream. The best confidence promoter match in each region was determined due the height of the FIMO score, strand direction and the position of the putative corresponding gene (Genbank RefSeq annotation) and/or previously reported RNA transcript [48]. Functional annotations and Gene-annotation enrichment analyses were done using DAVID Bioinformatics Resources (default settings) [49] and KEGG [50,51].

### Preparing of DNA templates

First, regions of interest were obtained from genomic DNA by PCR amplification with the Phusion DNA polymerase (New England Biolabs, Ipswich, USA) using corresponding primers (Additional file 1). For subcloning PCR products were ligated into SmaI and BamHI or HindII and BamHI double digested pUC19 plasmids and transformed into chemically competent *E. coli* DH5α cells. Correct plasmids were purified using the QIAprep plasmid isolation kit (Qiagen, Hilden, Germany) and used as PCR template for generating DNA templates used in *in vitro* assays. For EMSA assays templates were PCR amplified using corresponding primers (Additional file 1). One of the primers was labeled with 6-fluorescein (FAM). For all *in vitro* transcription assays DNA templates were PCR amplified using standard M13 forward and reverse primers. For DNaseI footprinting DNA templates were PCR amplified using the corresponding primers (Additional file 1).

### DNase I footprint

150 fmol template DNA and 0.3 μM TrmBL1 were incubated under the conditions used for the gel shift assay. 0.001 unit of DNase I was added for 1–6 min at 37 °C, and the reaction was stopped by the addition of 95 % formamide. DNA was ethanol precipitated and resuspended in 3 μl of formamide buffer. A DNA sequencing ladder using the same primer was generated as a molecular mass standard. Samples were loaded onto a 4.5 % denaturing polyacrylamide gel and analysed using an ABI PRISM 377 DNA sequencer.

### Electrophoretic mobility shift assay (EMSA)

150 fmol labeled DNA and various amounts of TrmBL1 were assembled in a 15 μl reaction volume according to Lee et al., 2008 [10]. After incubation at 70 °C for 15 min, protein-DNA complexes were analysed using a non-denaturating 6 % polyacrylamide gel. DNA fragments were visualized using a fluorimager.

### *In vitro* transcription assay

Assays were done as described previously [10,52]. Reaction mixtures with a total volume of 25 μl were assembled using transcription buffer supplemented with 95 nM TBP, 60 nM TFB, 11 nM RNAP, 150 fmol corresponding DNA template and various amounts of TrmBL1 as indicated. The final concentration of the NTP-mix in the reaction was 440 μM ATP, 440 μM CTP, 440 μM GTP, 2.7 μM UTP and [α-^32^P] UTP at 0.15 MBq (110 TBq/mmol). After incubation at 80 °C for 30 min the transcripts were separated on a denaturing 8 % polyacrylamide gel. Transcription products were visualized using a fluorimager.

## Results

A computational analysis revealed a very similar organization of the two transcriptional regulators TrmB and TrmBL1. Both proteins contain in the N-terminal region a highly conserved helix-turn-helix motif as DNA-binding-domain (45 % amino acid sequence identity) and an effector-binding domain in the C-terminal region [14,15]. To exclude the possibility that the close relationship of both proteins lead to a potential cross-reactivity of the polyclonal antibodies raised against TrmB (anti-TrmB IgG) and TrmBL1 (anti-TrmBL1 IgG) we started our analysis with western blot experiments.

**Fig. 1:**
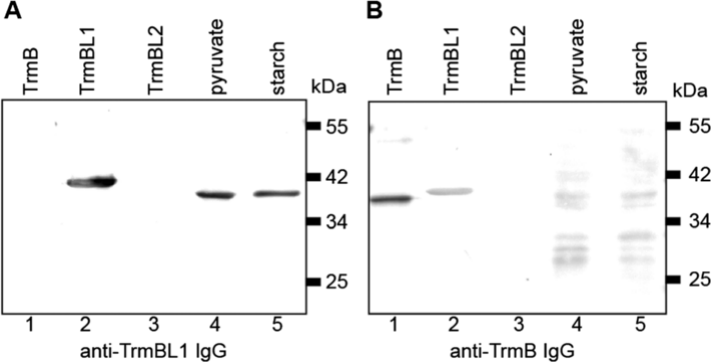
Western blot analysis using anti-TrmBL1 IgG and anti-TrmB IgG. 100 ng each recombinant TrmB (lane 1), TrmBL1 (lane 2) and TrmBL2 (lane 3) were used and 20 μg crude extract obtained from *P. furiosus* cells, which were grown under gluconeogenic (pyruvate, lane 4) or glycolytic (starch, lane 5) conditions. **a** using purified antibodies raised against recombinant TrmBL1 (anti-TrmBL1 IgG, 1:2000) for detection. **b** using purified antibodies raised against recombinant TrmB (anti-TrmB IgG, 1:2000) for detection

The anti-TrmBL1 IgG showed a specific reaction for recombinant TrmBL1 (Fig. 1a, lane 2) and no cross-reactivity with the paralogs TrmB and TrmBL2 (Fig. 1a, lane 1 and 3). Moreover, in crude extracts from cells grown under glycolytic (starch) or gluconeogenic (pyruvate) conditions one specific signal was detected (Fig. 1a, lane 4 and 5). As the recombinant TrmBL1 contains a His_6_ tag at the N-terminus, the electrophoretic mobility of the endogenous TrmBL1 was slightly reduced. In contrast, the antibody raised against TrmB showed a reaction with recombinant TrmB and a cross-reaction with TrmBL1 but not TrmBL2 (Fig. 1b, lane 1 to 3). In both crude extracts (starch or pyruvate) no specific signal for TrmB was detected (Fig. 1b, lane 4 and 5). Taken together, the anti-TrmBL1 IgG could be used for the ChIP experiments due to its high specificity, whereas the anti-TrmB IgG is not suitable due to the cross-reactivity to TrmBL1 and due to the missing specific signals in the crude extracts.

### TrmBL1 specifically binds various genomic loci under gluconeogenic growth conditions *in vivo*

**Fig. 2:**
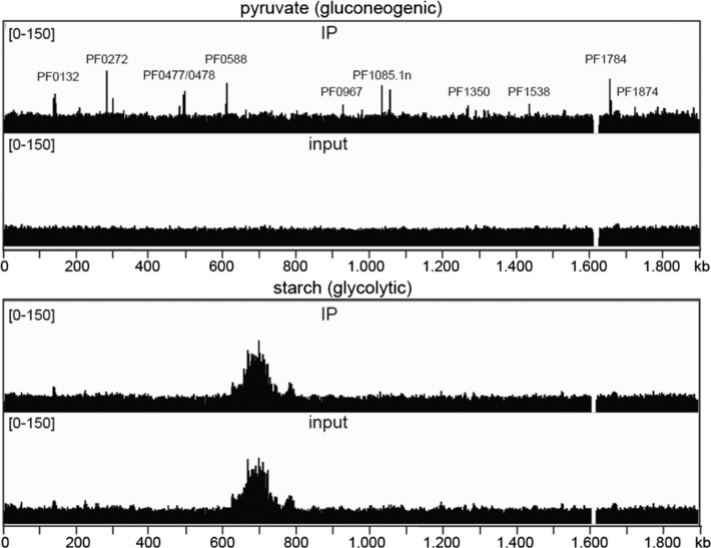
TrmBL1 binds to the genome under gluconeogenic growth conditions. TrmBL1 ChIP-seq experiments with *P. furiosus* cells grown under gluconeogenic (pyruvate 1) and glycolytic (starch 1) conditions were done and mapped TrmBL1 immunoprecipitation (IP 1) and input reads were visualized for the whole genome of *P. furiosus* using the IGV genome browser [67]. Prominent peaks found under gluconeogenic conditions are announced

TrmBL1 ChIP-seq experiments were performed using crude extracts from formaldehyde treated *P. furiosus* cells. Under gluconeogenic conditions two biological samples (pyruvate 1 and 2) were analysed and one under glycolytic conditions (starch 1). Figure 2 shows an overview of the mapped sequences within the complete *P. furiosus* genome under both conditions. Sequence analyses of all samples including inputs revealed two distinctive features: 1. The sequences contained nearby no hits in the published genome sequence from 1,613,140 to 1,629,427. This finding indicates that this part of the genome is deleted in the strain used for our experiments. 2. In the glycolytic input and immunoprecipitation samples we observed a strong accumulation in the genomic coverage of mapped reads from position 628,000 to 797,000.

Applying MACS2 we could identify in total 37 significantly enriched regions in the whole genome under gluconeogenic growth conditions (Fig. 2, Table 1 and Additional file 3). In contrast, under glycolytic conditions no significantly enriched sites were detected. This finding is consistent with earlier reports on TrmBL1 binding properties in the presence of certain sugars *in vitro* and *in vivo* [10,15,23].

**Table 1:** Selected known, predicted and novel TrmBL1 binding sites identified by ChIP-seq and corresponding genes

**Known and predicted TrmBL1 binding sites found by ChIP-Seq**				
Transcript organisation	**Gene**	**Gene product (Refseq)**	**Additional information**	**Position TGM relative to the promoter elements**
operon	**PF0132**PF0133	hypothetical protein hypothetical protein	putative isomaltase -	downstream
singleton	**PF0196**	glucose-6-phosphate isomerase	-	downstream
singleton	**PF0272**	alpha-amylase	-	downstream
singleton	**PF0464**	glyceraldehyde-3-phosphate: ferredoxin oxidoreductase	-	downstream
singleton	**PF0477**	alpha-amylase	cytoplasmatic	upstream
singleton	**PF0478**	alpha-amylase	-	downstream
singleton	**PF0588**	phospho-sugar mutase	-	downstream
operon	**PF1109**	hypothetical protein	one gene product: extracellular starch-binding protein	downstream
operon	PF1110	hypothetical protein		
singleton	**PF1784**	ADP-specific phosphofructokinase	-	downstream
singleton	**PF1874**	glyceraldehyde-3-phosphate dehydrogenase	-	upstream
singleton	**PF1920**	triosephosphate isomerase	-	downstream
operon	PF1933 PF1934 PF1935 PF1936 PF1937 **PF1938**	putative sugar transport ATP-hydrolyzing hypothetical protein amylopullulanase malG-like sugar transport inner membrane protein malF-like sugar transport inner membrane protein malE-like sugar binding protein	MD system	downstream
singleton	**PF1956**	fructose-bisphosphate aldolase	-	downstream
singleton	**PF1959**	cofactor-independent phosphoglycerate mutase	-	downstream
**Novel TrmBL1 binding sites found by ChIP-Seq**				
singleton	**PF0287**	pyrolysin	-	upstream
singleton	**PF0505**	hypothetical protein	predicted DNA binding protein	upstream
operon	PF0735	hypothetical protein	-	upstream
	**PF0736**	hypothetical protein		
singleton	**PF0853**	5'-methylthioadenosine phosphorylase	-	downstream
operon	**PF0874**	membrane dipeptidase	-	downstream
	PF0875	hypothetical protein	-	
operon	PF0965	pyruvate-ferredoxin oxidoreductase subunit beta	operon PF0971 to PF0965	downstream
	PF0966	pyruvate-ferredoxin oxidoreductase subunit alpha		
	**PF0967**	pyruvate-ferredoxin oxidoreductase subunit delta		
singleton	**PF1025**	hypothetical protein	conserved	downstream
singleton	**PF1062**	hypothetical protein	-	upstream
singleton	**PF1085.1n**	hypothetical protein	-	downstream
singleton	**PF1350**	major facilitator superfamily protein	transporter	downstream
singleton	**PF1476**	hypothetical protein	predicted transcriptional regulator: PadR family	downstream
operon	PF1535	alpha-glucan phosphorylase	same promoter region	downstream
	PF1536	hypothetical protein		
	PF1537	hypothetical protein		
	**PF1538**	N-ethylammeline chlorohydrolase		
singleton	PF1539	dihydroorotate dehydrogenase 1B		
singleton	**PF2016**	preprotein translocase subunit SecG	-	downstream
singleton	**PF2047**	l-asparaginase	-	upstream

Genes containing a TrmBL1 binding site in their promoter region are depicted in bold

### Validation of identified TrmBL1 binding sites *in vivo* by ChIP-qPCR

**Fig. 3:**
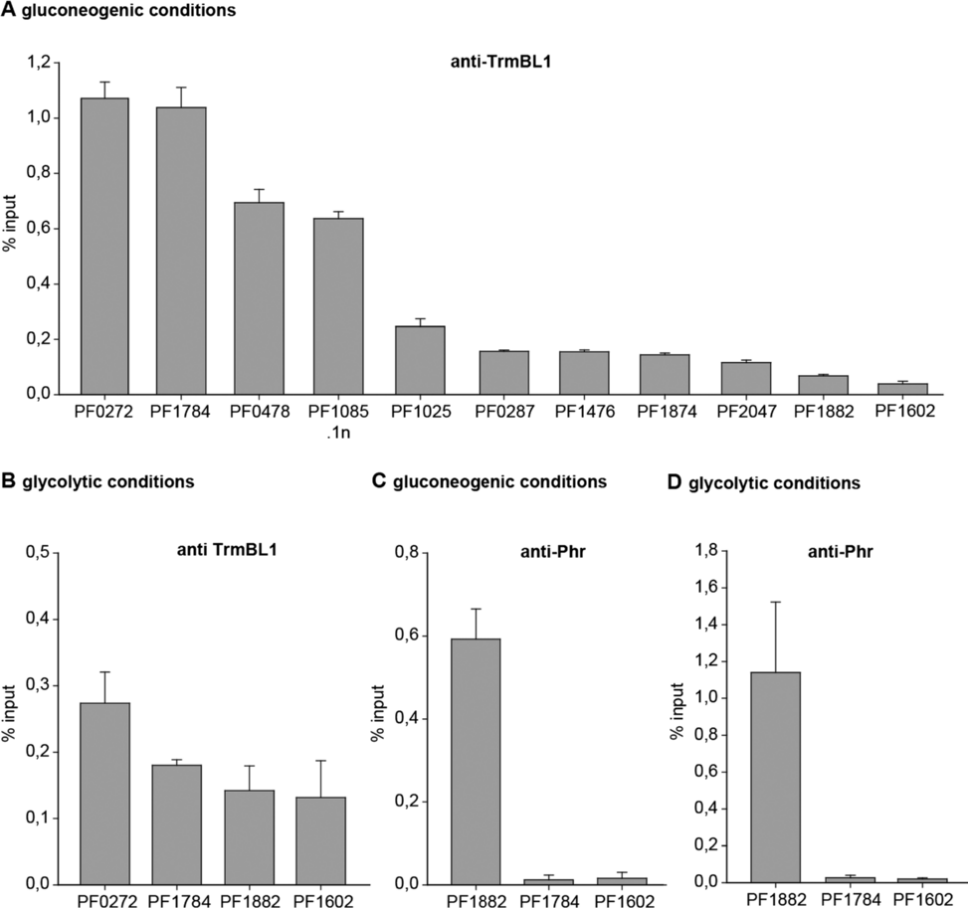
ChIP-qPCR validation of selected TrmBL1 binding sites identified by ChIP-Seq. ChIP enrichment is presented as % input. The mean with SD of at least three replicates of IP is shown for all analysed genomic loci. **a** TrmBL1 ChIP of cells grown under gluconeogenic condition (pyruvate 1). The genes PF1882 (*aaa + atpase*) and PF1602 (*gdh*) represent the negative controls for the anti-TrmBL1 IgG. **b** TrmBL1 ChIP of cells grown under glycolytic growth condition (starch 1). The genes PF1882 (*aaa + atpase*) and PF1602 (*gdh*) represent the negative controls for the anti-TrmBL1 IgG. **c** ChIP with a Phr specific antibody using cells grown on pyruvate (gluconeogenic conditions). The gene PF1882 (*aaa + atpase*; [29]) is the positive control for the anti-Phr IgG, whereas the genes PF1784 (*pfk*) and PF1602 (*gdh*) are the negative controls for the anti-Phr IgG. **d** ChIP with a Phr specific antibody using cells grown on starch (glycolytic conditions). The gene PF1882 (*aaa + atpase*) is the positive control for the anti-Phr IgG, whereas the genes PF1784 (*pfk*) and PF1602 (*gdh*) are the negative controls for the anti-Phr IgG

We used ChIP-qPCR assays as an alternative method to verify some of the sequence enrichments of the ChIP-seq experiments. The data were normalized using the percent input method and the promoter region of the *glutamate dehydrogenase* (*gdh*) gene (PF1602) as a negative control (Fig. 3a, b, c and d last row). The ChIP-qPCR data confirmed the specific ChIP-seq enrichments of all analysed putative TrmBL1 binding sites under gluconeogenic conditions (Fig. 3a and b). An antibody raised against the *Pyrococcus* heatshock regulator (Phr) served as negative control for specific enrichment using the TrmBL1 specific antibody for immunoprecipitation [5,30]. Using anti-Phr IgG no enrichment of the promoter region of the *phosphofructokinase* (*pfk*) gene was detected under both growth conditions (PF1784; Fig. 3c and d). In contrast, the previously identified Phr binding site in the promoter region of the *aaa + atpase* gene (PF1882) showed a strong ChIP-enrichment under both conditions. This demonstrates that the efficiency of formaldehyde crosslinking under both growth conditions was sufficient for successful ChIP experiments and that the absence of TrmBL1 binding events under glycolytic growth conditions using ChIP is specific for TrmBL1.

### TrmBL1 preferentially binds to TGM containing regions in the *P. furiosus* genome *in vivo*

**Fig. 4:**
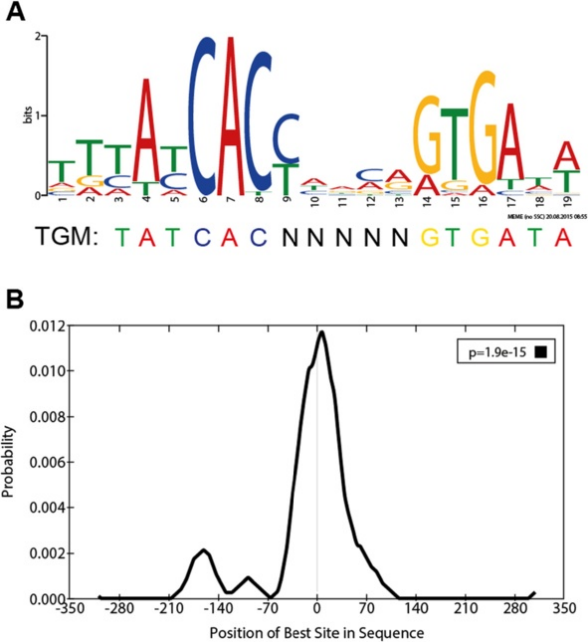
TrmBL1 preferentially binds to TGM containing regions in the *P. furiosus* genome. **a** Sequence logo (T [TG] [TC] A [TC] CAC [CT] [ATC] [AG] [CA] [AG] [GA] TGA [TA] [AT]); E-value = 1.1E-024) of the TGM based on 34 TrmBL1 binding sites identified by ChIP-seq and De-novo motif discovery using MEME. **b** Central motif enrichment of the TGM in the detected TrmBL1 binding sites (*E* –value = 1.9E-15; region width = 54) analysed by Centrimo

Previous studies showed that the TGM serves as palindromic DNA recognition element for TrmBL1 binding *in vitro* and *in vivo* [10,11,15,20]. Thus, identified TrmBL1 ChIP-enriched regions were analysed by the MEME suite [44]. De-novo motif discovery revealed the presence of a motif (T [TG] [TC] A [TC] CAC [CT] [ATC] [AG] [CA] [AG] [GA] TGA [TA] [AT]; E-value = 1.1E-24; Site Count = 34; Width = 19 nt) within 350 bps upstream and downstream in 34 of 37 TrmBL1 binding sites identified by ChIP-seq (Fig. 4a). This motif is quite similar to the previously reported TGM regarding length and consensus sequence TATCAC-N(5)-GTGATA [20]. Moreover, it is relatively enriched in the 37 peak regions compared with shuffled sequences (*P*-value = 5.44E-11; Wilcoxon rank-sum test; AME). Additionally, scanning for this consensus signature throughout the whole *P. furiosus* genome identified in total 70 motif occurrences with a *P*-value less than 1E-5 (FIMO). 57 % can be connected to 30 ChIP-enriched regions, whereas 43 % are associated with further 29 sites (Additional file 3). This suggests that TrmBL1 preferentially binds to TGM containing regions in the *P. furiosus* genome and may bind additional sites, which were not identified during our ChIP approach. Furthermore, local motif enrichment analysis showed that the TGM motif is centrally enriched in 68 % of the detected binding sites (Fig. 4b; *E* –value = 1.9E-15; region width = 54; region matches = 23; Centrimo). This indicates the high spatial resolution of the ChIP-seq approach, which was already described in previous reports [53].

### TrmBL1 binds downstream and upstream of promoter elements in the *P. furiosus* genome *in vivo*

TrmBL1 functions as transcriptional regulator which can act both as repressor and activator by binding downstream or upstream of the promoter elements [10,11,20]. 28 TrmBL1 binding sites (82 %) are in close proximity to promoters (~200 bp) of transcription units (single genes and operons), whereas six were found in transcribed regions (Additional file 4). For one site in the transcribed region of the gene PF2025 a strong promoter could be detected; however, no transcription unit could be identified. The TGM is mainly located directly downstream of the BRE- and TATA-box (75 %, 21 from 28 sites), which suggests transcriptional repression of these genes by inhibiting RNAP recruitment. The seven remaining motif occurrences are located up to 156 bps upstream of the corresponding promoter elements. The expression of these transcription units is presumably activated by TrmBL1 under gluconeogenic growth conditions. Additionally, a comparison with results from a microarray analysis of *P. furiosus* grown on carbohydrates or peptides showed that 13 of 20 transcription units harbouring a TGM downstream of the corresponding promoters are downregulated under gluconeogenic growth conditions, whereas six of seven transcription units containing a TGM upstream of their promoters are upregulated under gluconeogenic growth conditions (Additional file 4) [54]. Furthermore, TrmBL1 may act for one single promoter region both as repressor and activator. On the one hand it inhibits reverse transcription of the operon transcribed from PF1538 to PF1535. On the other hand it enhances forward transcription of the gene PF1539. A comparable transcriptional regulation mechanism was already shown for the archaeal sulphur response regulator SurR, which controls hydrogen and elemental sulfur metabolism in *P. furiosus* [9].

### ChIP-seq experiments identified known and novel TrmBL1 binding sites

Binding 28 promoter regions in the *P. furiosus* genome extends the TrmBL1 regulon to up to 43 genes (single genes and operons; Table 1, Additional file 4 and 5). Some of the corresponding genes displaying strong ChIP-enrichment signals in their upstream regions are labeled in Fig. 2 (lane 1). Functional annotations of the 43 genes were done using KEGG. Gene-annotation enrichment analysis by DAVID regarding the gene ontology (GO) term biological process (GOTERM_bp_all) revealed significant enrichment of 16 GO terms (EASE threshold: 0.1 and Count: 2), which are mainly related to sugar uptake, glycolysis and gluconeogenesis e.g. GO0016052 (carbohydrate catabolic process), GO0005975 (carbohydrate metabolic process) and GO0006096 (glycolysis). All found enriched GO terms are shown in Additional file 6. Most important, the corresponding genes, which contain a TrmBL1 binding site in their promoter region, were already studied by *in vitro* and/or *in vivo* analysis or predicted as target of TrmBL1 mediated transcriptional control due to the presence of the TGM by *in silico* analysis (Table 1; Known and predicted TrmBL1 binding sites) [10,11,15,20]. This includes several genes encoding possible α-glucan hydrolyzing enzymes (PF0272, PF0477, PF0478 and PF1935), which may function at various steps of starch degradation to glucose [55]. Moreover, the ChIP-seq experiments confirmed that TrmBL1 *in vivo* plays a role in the transcriptional control of the previously analysed MD operon (PF1938-PF1933); [15,56]. Further glycolytic-specific genes regulated by TrmBL1 encode for example the glyceraldehyde-3-phosphate ferredoxin oxidoreductase (gapor; PF0464) and the ADP-specific phosphofructokinase (pfk, PF1784). In contrast, the *glyceraldehyde-3-phosphate dehydrogenase* (*gapdh*, PF1874) seems to be the only gene which exhibits promoter bound TrmBL1 under gluconeogenic conditions and which most likely plays a role in gluconeogenesis instead of glycolysis [16].

In addition to the known binding sites, our ChIP-seq analysis also revealed 14 novel TrmBL1 binding sites in the genome of *P. furiosus* (Table 1; Novel TrmBL1 binding sites). Most of these newly identified genes seem to be not directly involved in the regulation of sugar uptake, glycolysis or gluconeogenesis. The finding that TrmBL1 also binds the upstream regions of two predicted transcriptional regulators containing helix-turn-helix DNA binding motifs (PF0505 and PF1476) indicates that TrmBL1 could also act as a more global regulator. Additional genes harbouring a TrmBL1 binding site in their upstream regions were the *L-asparaginase* (PF2047) or a transmembrane transporter of the major facilitator superfamily (MFS; PF1350) and various genes encoding hypothetical proteins with unknown function (e.g. PF0736, PF1025, PF1085.1n). One newly identified binding site under gluconeogenic conditions is located upstream of the *pyruvate-ferredoxin oxidoreductase* (*por*) δ subunit gene (PF0967), which is part of a polycistronic operon transcribed from PF0971 to PF0965 [57]. Furthermore, two novel TrmBL1 sites were detected in the promoter regions of the genes encoding *pyrolysin* (PF0287) and a *membrane dipeptidase* (PF0874), which are participating in proteolysis.

### Validation of identified TrmBL1 binding sites *in vitro*

**Fig. 5:**
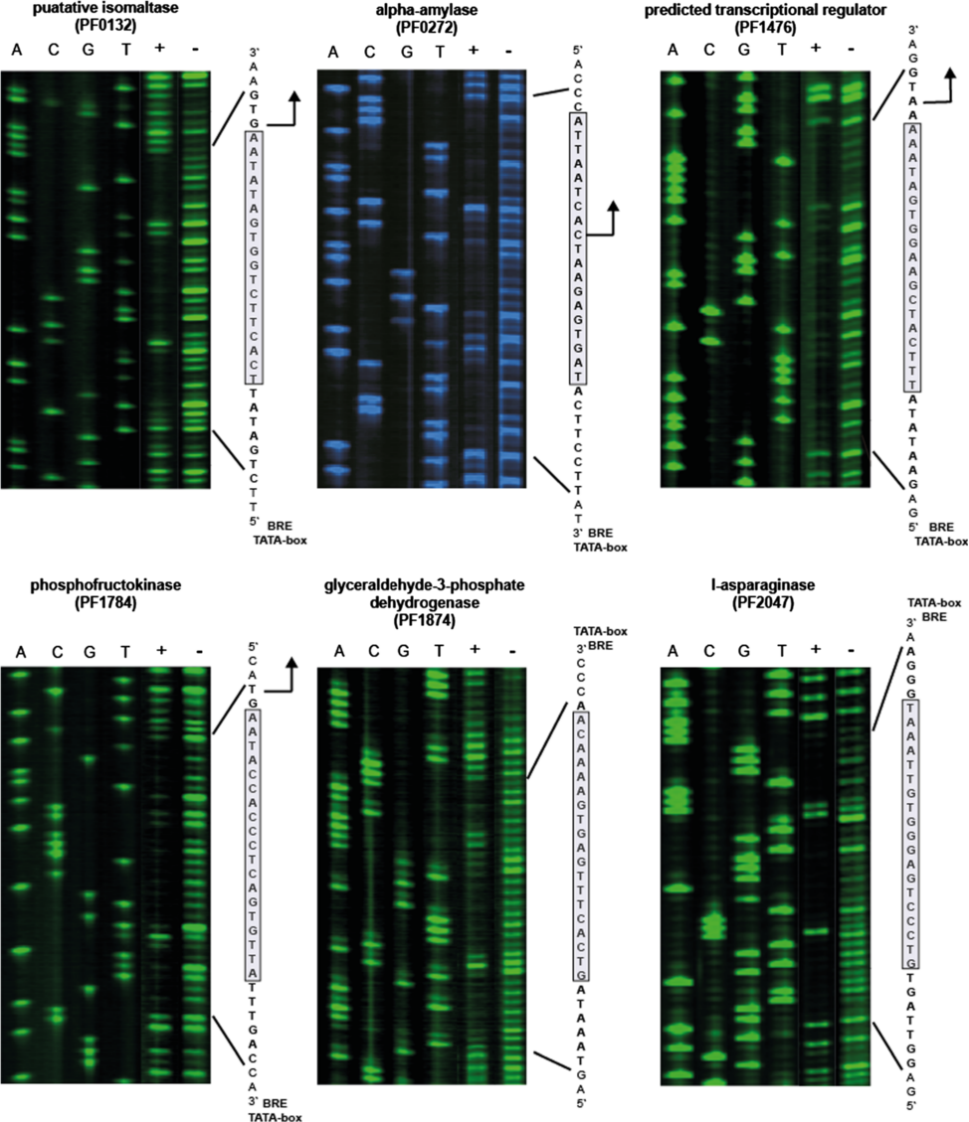
TrmBL1 DNase I footprint regions enclose the TGM. DNaseI footprints were done in the presence (+) or absence (−) of TrmBL1 using various identified binding sites. DNA ladders and orientations of the used template strands (transcribed or non-transcribed) are marked by 5’ and 3’. TrmBL1 footprint regions are written in bold letters and the TGMs are highlighted as grey box. Translational start sites, if present, are shown as black arrow and positions of the BREs and TATA-boxes are announced

**Fig. 6:**
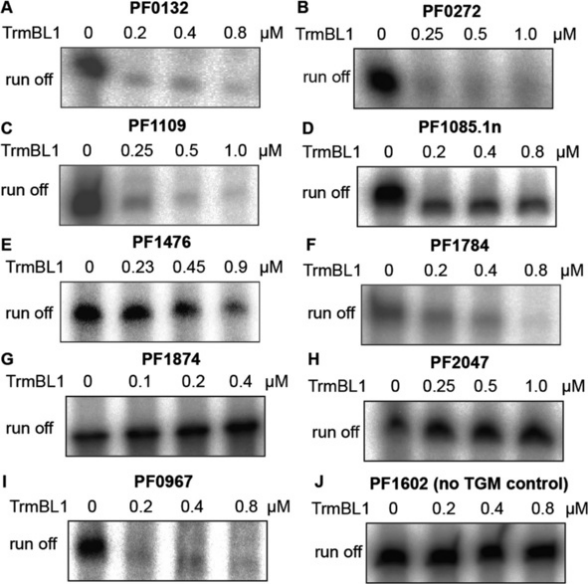
TrmBL1 functions as transcriptional repressor and activator. *In vitro* transcription assays were done using DNA templates containing the TGM downstream or upstream of the promoter (A -I). The concentrations of TrmBL1 were indicated on top of each lane. Template PF1602 contains the *gdh* promoter without TGM as a negative control (J).

Our ChIP-seq approach revealed 28 TrmBL1 binding sites in the genome of *P. furiosus*, which contain the TGM and which are located in close proximity to promoter regions. First of all, EMSAs were carried out to confirm specific binding of TrmBL1 to 15 selected putative binding regions (nine known and seven novel binding sites). These EMSA experiments clearly demonstrated binding of TrmBL1 to all tested templates (Additional file 7 A to O). In contrast, no shift could be detected using the *gdh* promoter region as template, which contains no TGM (Additional file 7 P). Moreover, the ChIP-seq data indicates a close relationship between the identified binding positions and the presence of the TGM, which was already suggested by previous reports [10,11,15,20]. Thus, DNase I footprinting experiments were performed to verify the function of the TGM as cis-regulatory DNA element for TrmBL1 binding. In all six analysed promoter regions the TrmBL1 footprint protects the TGM sequence (Fig. 5). Additionally, the results of our ChIP-seq approach suggest that due to its high spatial resolution in combination with detailed motif analysis it enables predictions about the regulation mode of TrmBL1 as transcriptional repressor (binding downstream of the promoter elements) or activator (binding upstream of the promoter elements). For verification of this assumption we used 12 templates in *in vitro* transcription experiments. In the presence of increasing concentrations of TrmBL1 the transcription of all nine templates was repressed where the binding site is located downstream of the promoter (Fig. 6a to f and Additional file 8 A, C and D). In contrast, the transcription of the two templates containing the TGM located upstream of the BRE and TATA-box were activated in the presence of TrmBL1 (Fig. 6g and h). Only for one template harbouring the TGM located upstream of the promoter no effect on transcription could be detected in the presence of TrmBL1 (Additional file 8 B). Furthermore, *in vitro* transcription experiments using the upstream sequence of the *por* δ subunit (PF0967) confirmed the presence of an additional promoter within the polycistronic operon of PF0971 to PF0965 (Fig. 6i). This internal promoter is repressed under gluconeogenic conditions due to binding of TrmBL1. A negative control template without TGM confirmed the requirement of the cis element for transcriptional regulation (Fig. 6j). In summary, the experimental confirmation *in vitro* revealed that our ChIP-seq approach allows a precise determination of the TrmBL1 binding sites which enabled reliable predictions about the regulation mode of TrmBL1 at almost all genes.

### Deletion of the 16 kb fragment includes TrmB

**Fig. 7:**
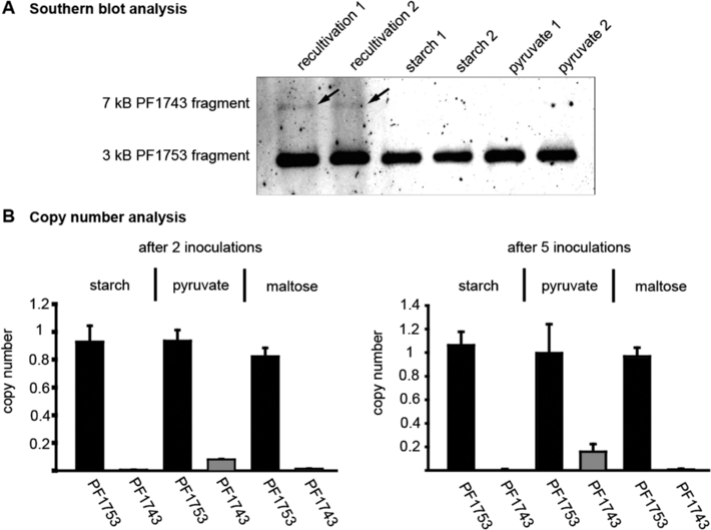
Deletion of a 16 kb fragment encoding the TM system and TrmB. **a** southern blot analysis using BamHI and SmaI digested *P. furiosus* genomic DNA. DNA was isolated from cells after recultivation and adaption to glycolytic (starch) or gluconeogenic (pyruvate) growth conditions. The result of two independent recultivations is shown. The probe, specific for PF1753, detects a 3 kb fragment, while the PF1743 specific probe binds to a 7 kb fragment. **b** copy number analysis of the genes PF1743 and PF1753 in *P. furiosus* cells grown under the following conditions: glycolytic (starch and maltose) or gluconeogenic (pyruvate). The gene PF1784 was used as calibrator and the recultivation culture as reference. The mean of three biological replicates including SD is shown

As mentioned above mapping of the sequenced reads revealed a deletion of a 16 kb fragment from gene PF1737 to PF1751 (Fig. 2, chromosomal position 1,613,140 to 1,629,427). This fragment contains the TM system (PF1739 to PF1747), which encodes for the trehalose/maltose-specific-ABC transporter [56]. The transcriptional regulator TrmB is also part of this operon and therefore it was not possible to detect the corresponding signal in western blot experiments using *P. furiosus* cell extracts (Fig. 1b, lane 4 and 5). To exclude the possibility that the deletion of this fragment is only a characteristics of our strain we ordered a new one from the DSMZ. The fragment is present in the new strain, but a southern blot analysis revealed that the fragment disappeared after a few transfers into fresh medium (Fig. 7a). The initial re-cultivation culture exhibited only a weak signal for the *trmB* fragment. This indicates that most of the genomes harbour already the deletion. To study the loss of this fragment in more detail a copy number analysis was performed after two and five transfers into different media (Fig. 7b). While under glycolytic conditions after two passages the fragment is nearby completely deleted (<0.05), the copy number under gluconeogenic conditions varies between 0.15 after two inoculations and 0.20 after five inoculations. Even growth on maltose could not prevent deletion of this fragment.

## Discussion

Initially, we planned to study the function of the two paralogous proteins TrmB and TrmBL1 from *P. furiosus* in a genome-wide manner by ChIP-seq *in vivo*, but the cross-reactivity of anti-TrmB antibody with TrmBL1 and the discovered 16 kb deletion including *trmb* in the *P. furiosus* genome inhibited this approach. The deleted fragment contains the complete TM operon, which also encodes for a trehalose/maltose-specific-ABC-transporter. The deleted part belongs to one of six highly variable chromosomal regions, which were previously described in a population of *Pyrococcus* isolates from Vulcano Island, Italy [58]. Furthermore, this 16 kb fragment is flanked by two insertion sequence (IS) elements and proposed as an example for a recent transposon mediated gene transfer between *P. furiosus* and *Thermococcus litoralis* [59,60].

The analysis of a new type strain ordered from the DSMZ indicated that this fragment was almost completely deleted after the first re-cultivation and even growth on maltose could not prevent the deletion of the fragment (Fig. 7). From previous data it is known that growth on maltose also induces the expression of the MD operon [56]. These authors assume that the used maltose in the medium is contaminated with maltotriose. We have used different amounts of maltose, but only a very high concentration (5 % v/v) of maltose enabled the growth of *P. furiosus*. This finding also argues for a contamination with long-chained sugars and in consequence there is no possibility to exert selection pressure for keeping the TM operon. The reason why this fragment seems to become deleted even in the first re-activation culture is unknown.

Focusing on ChIP-seq analysis of TrmBL1 revealed 37 putative binding sites in the *P. furiosus* genome and the presence of the TGM could be shown for 34 identified regions. This also confirmed previous data suggesting this palindromic DNA motif as exclusive binding site of this regulator [10,11,15,20]. Six binding sites are located in transcribed regions far from any translational start site and/or promoter. The function of these sites regarding transcriptional control remained unclear. However, the identification of binding sites within transcribed regions was already reported for three archaeal TFs including TrmB from *H. salinarium* NRC-1 [23,25,26]. In contrast, 28 identified binding sites are in close proximity of promoter regions of single genes and operons.

Beside 14 well-known binding targets of TrmBL1, the ChIP-seq approach also enabled the identification of 14 new binding sites in the P. *furiosus* genome. This extends the TrmBL1 regulon to up to 43 genes. The corresponding newly identified genes include a putative membrane transport protein of the MFS (PF1350), which may play a role in sugar uptake. In addition, the identified binding sites upstream of PF0967 and PF1476 pointed to a higher-ranking influence of TrmBL1 on transcriptional regulation: The first one is located within an operon transcribed from PF0971 to PF0965 [48], but an additional separate transcript of PF0967 to PF0965 has been also suggested for this region [57,61]. PF0967 to PF0965 encode three subunits of the POR and PF0970 to PF0968 encode three subunits of the ketoisovalerate ferredoxin oxidoreductase (VOR). Both enzymes were heterotetramers and the missing γ-subunit is encoded by the first gene PF0971 of the operon and is shared by both enzymes [57,61]. *In vitro* experiments confirmed that the binding of TrmBL1 represses transcription starting from the promoter upstream of PF0967 (Fig. 6 i). Under gluconeogenic growth conditions this would cause less conversion of pyruvate to acetyl-CoA and increase the amounts of available pyruvate for gluconeogenesis. It is interesting to note that the available microarray data neither from *Pyrococcus* nor from *Thermococcus* indicate this type of regulation [11,54]. Additional promoters within archaeal operons and their implications for modulating different responses according to the environmental challenge has already been shown for *H. salinarum NRC-1* [62].

The second newly identified binding site is upstream of PF1476. The corresponding protein belongs to the PadR-family which regulates phenolic acid decarboxylase in bacteria [63]. The function in Archaea is unknown. It is interesting to note that transcriptional repression of PF1476 by TrmBL1 is conserved between *P. furiosus* and *T. kodakarensis* [11]. Elucidation of the role of this regulator will be a straight forward task for the future to completely understand the role of TrmBL1 as a superordinate player in gene regulation. This is also encouraged by the finding that TrmBL1 activates expression of a second gene (PF0505) containing a HTH motif, which suggests a presumable function as transcriptional regulator.

Previous *in vitro* studies indicated that the cellular concentration of TrmBL1 is controlled by autoregulation [15]. But under the tested conditions we could not identify binding of TrmBL1 upstream of its own gene. In contrast to *T. kodakarensis* the *trmBL1* promoter has no TGM and we assume that binding of TrmBL1 to the TGM and its function as transcriptional regulator in *P. furiosus* is mainly controlled by the presence of inducers and possible co-repressors. The promoter-dependent function of various inducers was already demonstrated by *in vitro* transcription experiments [10,11,15].

The ChIP-seq data also revealed that some of the identified binding sites are located upstream of the promoter. A previous *in vitro* study already reported that binding of TrmBL1 under gluconeogenic conditions upstream of the promoter leads to transcriptional activation [10]. Thus, the identification of TrmBL1 binding upstream of the promoter of the *glycerin aldehyde phosphate dehydrogenase* gene (PF1874) and the *l-asparaginase* (PF2047) and experimental confirmation is in perfect agreement with published data [11,54]. In both cases, the expression under gluconeogenic conditions is increased, but so far no mechanistic details about the activation mechanism are known. It is possible that TrmBL1 acts in a similar mode as the transcriptional activators Putative transcriptional regulator 2 (Ptr2) or TFB recruiting factor 1 (TFB-RF1). The first one facilitates the recruitment of TBP and the second one promotes TFB binding [6,7].

**Fig. 8:**
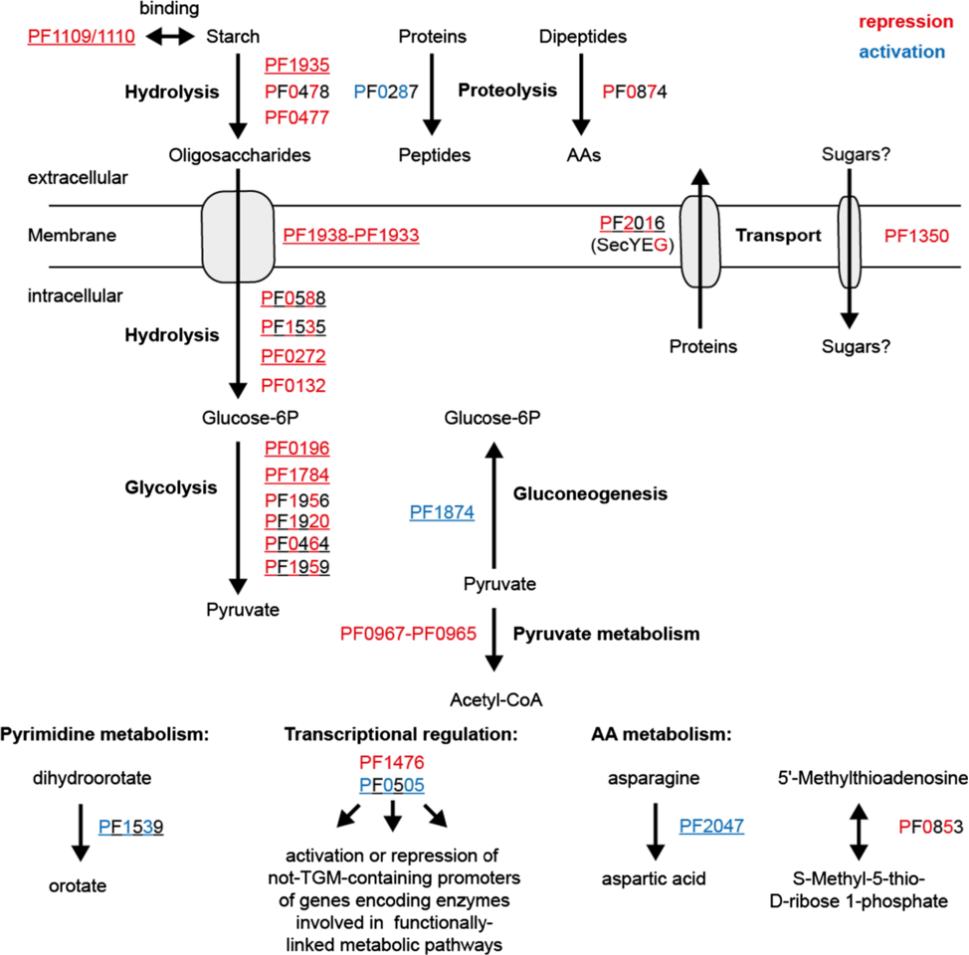
TrmBL1 controls expression of genes involved in functionally-linked metabolic pathways by its own or in concert with secondary regulators. TrmBL1 mediated repression or activation is depicted in red or blue. Complete colouring indicates validation of repression or activation by cell-free transcription. Banded colouring indicates suggested transcriptional effects. Genes differentially expressed in a microarray analysis of *P. furiosus* grown on maltose or peptides are underlined [54]. Gene products: PF0132, proposed α-glucosidase; PF0196, phosphoglucose isomerase; PF0272, proposed 4-α-glucanotransferase; PF0287, pyrolysin; PF0464, glycerinaldehyde 3-phosphate:ferredoxin oxidoreductase; PF0477, proposed extracellular α-amylase. PF0478, proposed extracellular cyclomaltodextrin glucano-transferase; PF0505, putative DNA binding protein; PF0588, phosphoglucose mutase; PF0853, 5'-methylthioadenosine phosphorylase; PF0874, membrane dipeptidase; PF0967-PF0965, pyruvate ferredoxin oxidoreductase; PF1109/1110, extracellular starch-binding protein; PF1350, proposed single-component transport protein; PF1476, putative PadR-like regulator; PF1535, maltodextrin phosphorylase; PF1539, dihydroorotate dehydrogenase 1b; PF1784, phosphofructokinase; PF1874 glycerinaldehyde 3-phosphate dehydroghenase; PF1920, triosephosphate isomerase; PF1935, amylo-pullulanase; PF1938-PF1933 maltodextrin-specific ABC transporter (MD-system); PF1956 phosphoglycerate mutase; PF1959, fructose-1,6-bisphosphate aldolase; PF2016, preprotein translocase subunit SecG; PF2047, L-asparaginase. AA; amino acid

Unexpectedly, no binding of TrmBL1 to the promoter region of the *fructose-1,6-bisphosphatase* gene (*fbp*; PF0613) was detected in our study. Previous results showed TrmBL1 mediated activation of the transcription of this gene for *P. furiosus* and *T. kodakarensis* [10,11]. Additionally, the FIMO search for TGM occurrences in the whole *P. furiosus* genome confirmed the previously reported presence of a corresponding motif sequence in the PF0613 promoter region (Additional file 3) [20]. However, there are several additional putative TrmBL1 binding sites, which also appear to be unbound by TrmBL1 *in vivo* using ChIP-seq. The binding of TrmBL1 not only relies on the presence of the TGM. Previous reports indicate that the function of TrmBL1 is also impaired by the presence of certain sugars, which act as inducers or possible co-repressors (see above). Gene expression microarray analyses of *P. furiosus* cells, which were grown on a variety of glucans, revealed differential transcriptional responses to this different carbon sources [55,64]. This includes several genes (e.g. PF2047), whose expression is regulated by TrmBL1. Thus, we suggest that equivalent varying transcriptional responses can also be observed using pyruvate or peptides as carbon source. This may also explain the deviations between the ChIP-seq results and the microarray analysis of *P. furiosus* grown on carbohydrates or peptides [54]. For the microarray analysis *P. furiosus* was grown in the presence of S^0^ with peptides for gluconeogenic and with maltose for glycolytic conditions. For the ChIP-seq experiment *P. furiosus* was grown in the absence of S^0^ with sodium pyruvate for gluconeogenic and with starch for glycolytic conditions. Furthermore, the methods are different: ChIP-seq gives a picture about TrmBL1 molecules bound to DNA and the microarray experiment provides information about quantified RNA molecules. Nevertheless, the ChIP-seq technique complements the microarray data and it increases the number of available global *in vivo* methods for the hyperthermophilic euryarchaeon *P. furiosus* to study gene regulatory networks in more detail. In the case of the TrmBL1 network it enabled confirmation of known targets of TrmBL1 mediated transcriptional control. The corresponding genes encode enzymes which participate in sugar uptake, glycolysis and gluconeogenesis. Furthermore, the identification of novel binding sites and the experimental verification clearly indicate that TrmBL1 is also involved in regulation of genes encoding enzymes involved in additional cellular processes e.g. proteolysis, amino acid metabolism, transcriptional control or metabolic pathways. The TrmBL1-regulated targets obtained from ChIP-seq are summarized in Fig. 8. These data indicate that TrmBL1 is also a more global regulator similar to the function of TrmB in *H. salinarium* NRC-1. In this case TrmB plays an important role for sensing disposability of a carbon source and controlling the appropriate transcriptional response by its own or together with additional regulators [23,65,66].

## Conclusions

While ChIP-seq became a widely used approach to study genome-wide protein-DNA interactions in bacterial and eukaryotic model organisms in vivo, it has been hitherto rarely applied in archaeal systems. Our ChIP-seq analysis of TrmB and TrmBL1 increased the number of available in vivo approaches to analyze gene regulatory networks in P. furiosus, which represents beside T. kodakarensis one widely used archaeal model organism within the order Thermococcales. In concert with the well-established in vitro assays of the P. furiosus transcription system, it represents a powerful tool, which can not only help to understand regulation of gene expression, but also to dissect the underlying mechanisms. This technique lead to two major findings: A, in the used P. furiosus strain `DSM3638´ a 16 kb fragment harboring the TM-system including TrmB was deleted. Thus, function of TrmB in regulation of sugar metabolism could not be studied using this strain; B, In contrast, using ChIP-seq for mapping of TrmBL1 binding sites in a genome wide manner for P. furiosus in vivo revealed an extended function of TrmBL1 as global regulator. TrmBL1 not only regulates genes involved in sugar metabolism, but it also controls transcription of genes involved in various additional metabolic pathways and biological processes as proteolysis or the amino acid metabolism 

### Availability of supporting data

ChIP-seq raw data are available in the ArrayExpress database (www.ebi.ac.uk/arrayexpress) under accession number E-MTAB-3959. Further data are included as additional files.

## Additional files

Additional file 1:
**Used primer sequences and plasmids.** (XLSX 24 kb) Additional file 2:
**Fragmentation of** ***P. furiosus*** **genomic DNA by sonication.** (PDF 1040 kb) Additional file 3:
**Position of TrmBL1 binding sites and occurrences of the** ***Thermococcales*** **Glycolytic Motif (TGM) in the** ***P. furiosus*** **genome.** (XLSX 19 kb) Additional file 4:
**Position of the TrmBL1 binding sites in relation to annotated genes, RNA transcripts and promoter regions and differential expression of these genes under glycolytic versus gluconeogenic growth conditions.** (XLSX 23 kb) Additional file 5:
**Functional annotation of genes of the TrmBL1 regulon.** (XLSX 15 kb) Additional file 6:
**Gene-annotation enrichment analysis.** (XLSX 13 kb) Additional file 7:
**EMSAs with TrmBL1 and 15 identified binding regions.** (PDF 892 kb) Additional file 8:
***In vitro*** **transcription assays.** (PDF 978 kb)

## Abbreviations

TF: Transcription factor
ORF: Open reading frame
TrmB: Transcriptional regulator of mal operon
TrmBL1: TrmB-like protein 1
BRE: TFB recognition element
TBP: TATA binding protein
TFB: transcription factor B
TGM: Thermococcales glycolytic motif
TM: trehalose and maltose specific ABC transporter
RNAP: RNA polymerase
ChIP: Chromatin immunoprecipitation
DSMZ: Deutsche Sammlung von Mikroorganismen and Zellkulturen
qPCR: quantitative real-time PCR
Cq: quantification cycle
SD: standard deviation
IP: immunoprecipitation
MACS: Model-based analysis for ChIP-seq
pfk: phosphofructokinase
gapor: glyceraldehyde-3-phosphate ferredoxin oxidoreductase
gapdh: glyceraldehyde-3-phosphate dehydrogenase
MFS: Major facilitator superfamily
por: pyruvate-ferredoxin oxidoreductase
IS: Insertion sequence
vor: ketoisovalerate ferredoxin oxidoreductase
Ptr2: Putative transcriptional regulator 2
TFB-RF1: TFB recruiting factor 1
fbp: fructose-1, 6-bisphosphatase
MD: Maltodextrin-specific ABC transporter
