# Supplementary material for: Genome-wide binding analysis of the transcriptional regulator TrmBL1 in Pyrococcus furiosus
Source: BMC Genomics. 2016 Jan 8;17:40. doi: 10.1186/s12864-015-2360-0 (PMC4706686; doi:10.1186/s12864-015-2360-0)
Supplement: Additional file 2: — Fragmentation of P. furiosus genomic DNA by sonication. (PDF 1040 kb) [file 12864_2015_2360_MOESM2_ESM.pdf]

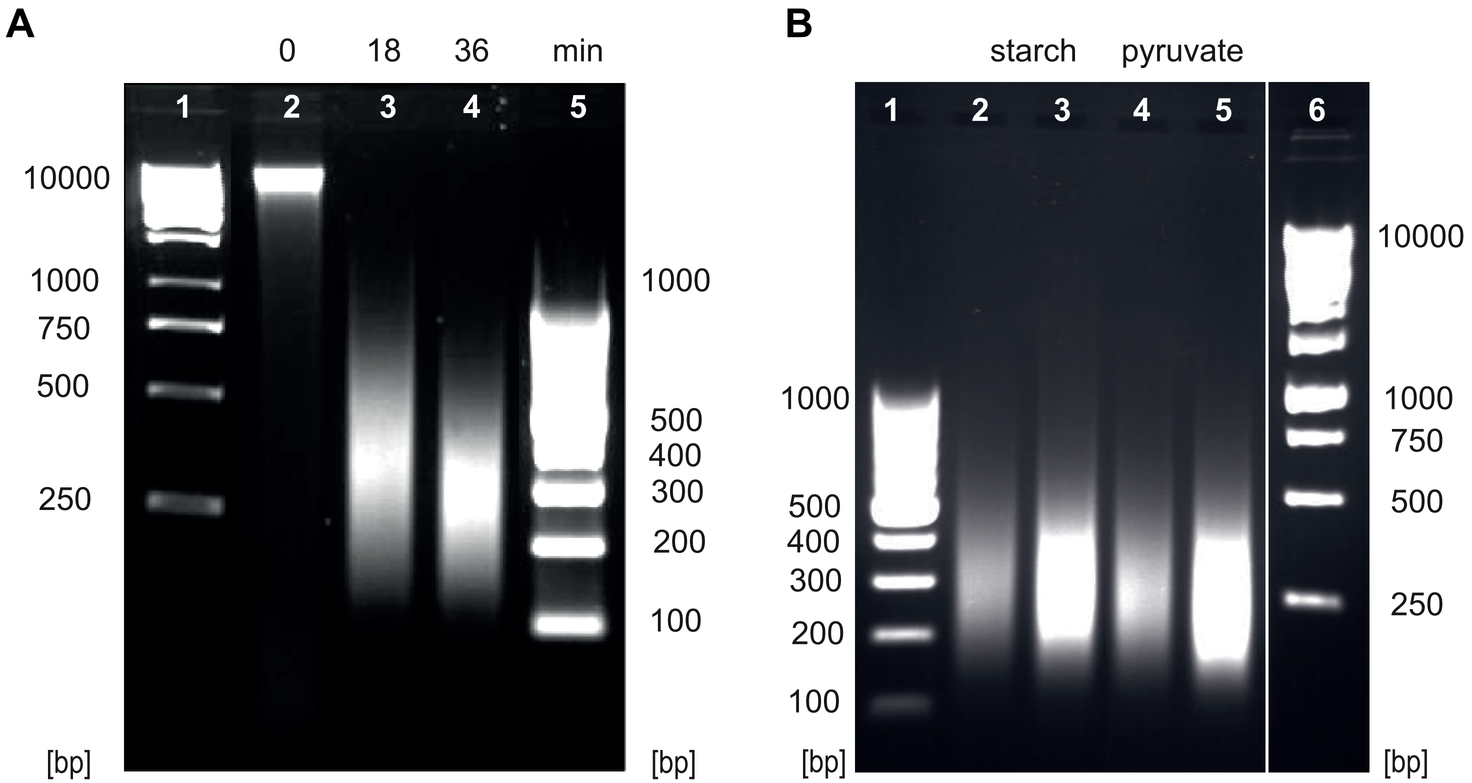

Additional file 2. Fragmentation of *P. furiosus* genomic DNA by sonication. A, time course of sonication of formaldehyde treated *P. furiosus* cells, grown on complex media. Crosslink was reversed over night and fragmented DNA was analyzed by a 1.5% agarosegel. 1, 1kb ladder; 2, after no sonication; 3, after sonication for 18 min (total time); 4, after sonication for 36 min (total time); 5, 100 bp ladder. B, sonication of formaldehyde-treated *P. furiosus* cells grown under glycolytic (starch 1) or gluconeogenic (pyruvate 1) conditions. Sonication was done for 36 min (total time). After reversal of the crosslink and purification various amounts of DNA were analyzed on a 1.5 % agarosegel. 1, 100 bp ladder; 2, 200 ng fragmented DNA (starch 1); 3, 400 ng fragmented DNA (starch 1); 4, 200 ng fragmentedDNA (pyruvate 1); 5, 400 ng fragmented DNA (pyruvate 1); 6, 1 kb ladder.
