# Supplementary material for: Genome-wide binding analysis of the transcriptional regulator TrmBL1 in Pyrococcus furiosus
Source: BMC Genomics. 2016 Jan 8;17:40. doi: 10.1186/s12864-015-2360-0 (PMC4706686; doi:10.1186/s12864-015-2360-0)
Supplement: Additional file 7: — EMSAs with TrmBL1 and 15 identified binding regions. (PDF 892 kb) [file 12864_2015_2360_MOESM7_ESM.pdf]

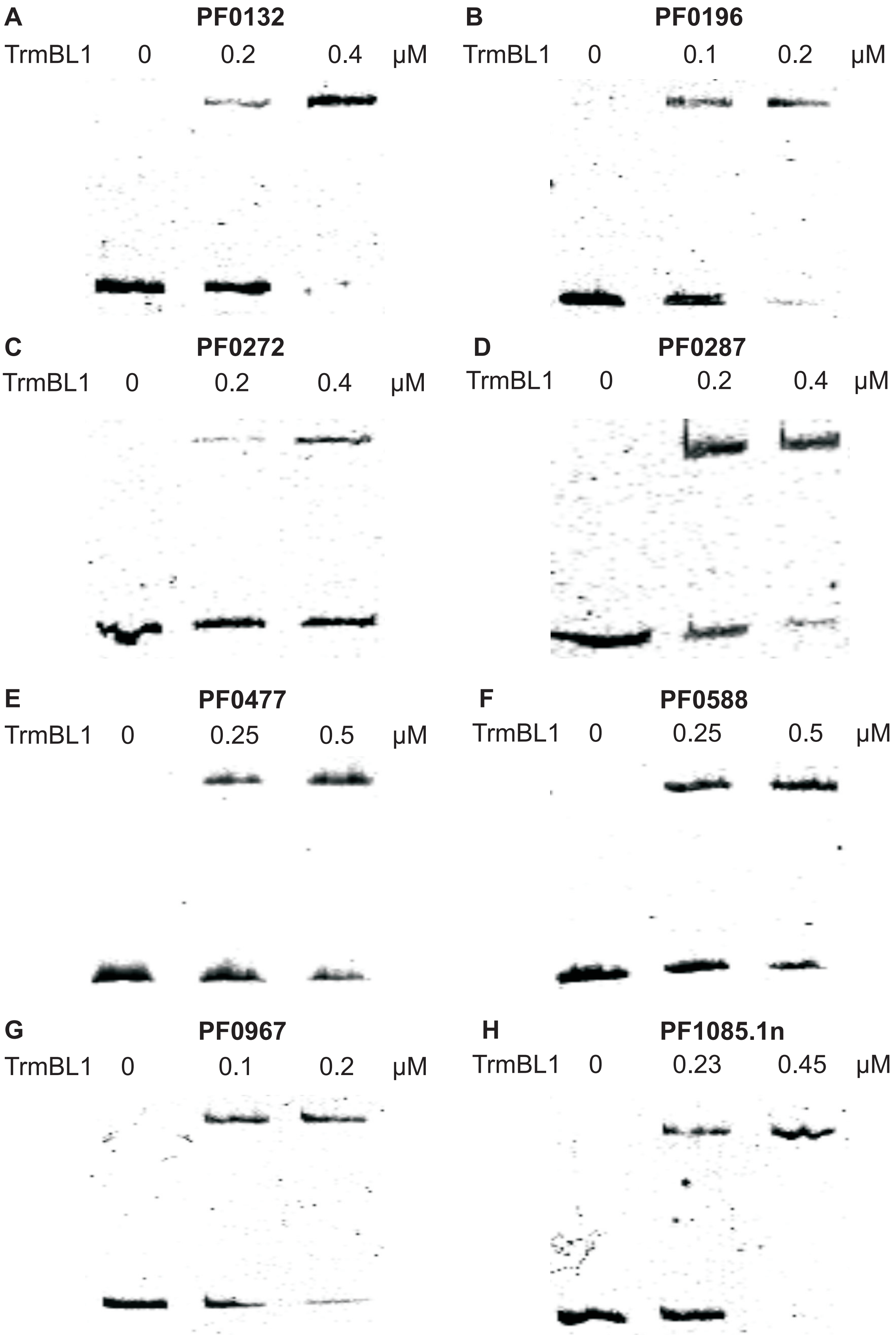
A
PF0132
TrmBL1 0 0.2 0.4 µM
B
PF0196
TrmBL1 0 0.1 0.2 µM
C
PF0272
TrmBL1 0 0.2 0.4 µM
D
PF0287
TrmBL1 0 0.2 0.4 µM
E
PF0477
TrmBL1 0 0.25 0.5 µM
F
PF0588
TrmBL1 0 0.25 0.5 µM
G
PF0967
TrmBL1 0 0.1 0.2 µM
H
PF1085.1n
TrmBL1 0 0.23 0.45 µM

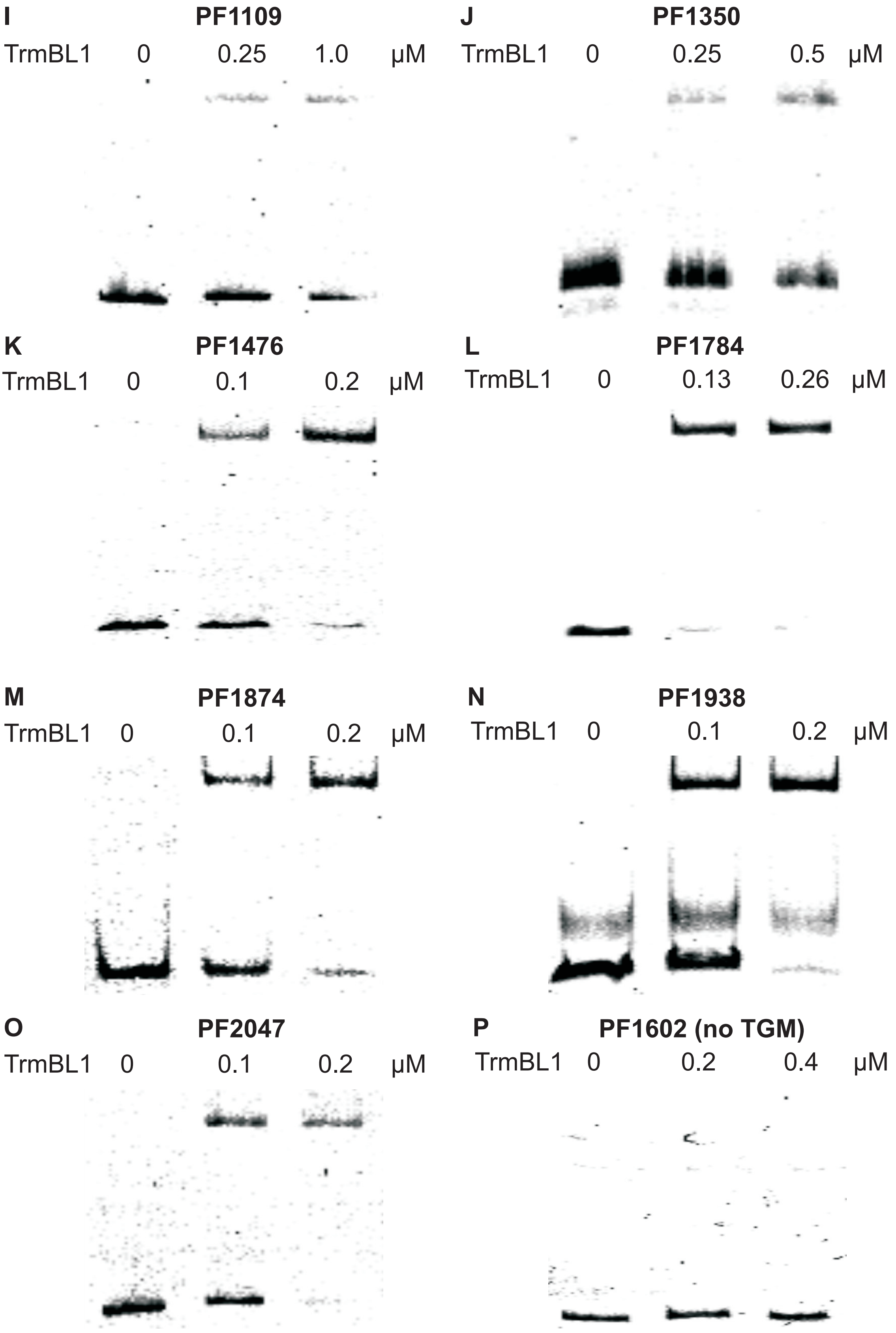

Additional file 7. EMSAs with TrmBL1 and 15 identified binding regions. EMSAs were performed without or with increasing TrmBL1 concentrations as indicated on top of the lanes. The templates were amplified using PCR with labelled primers. The sequence of the primer is indicated on additional file 2.
