# Supplementary material for: Genome-wide binding analysis of the transcriptional regulator TrmBL1 in Pyrococcus furiosus
Source: BMC Genomics. 2016 Jan 8;17:40. doi: 10.1186/s12864-015-2360-0 (PMC4706686; doi:10.1186/s12864-015-2360-0)
Supplement: Additional file 8: — In vitro transcription assays. (PDF 978 kb) [file 12864_2015_2360_MOESM8_ESM.pdf]

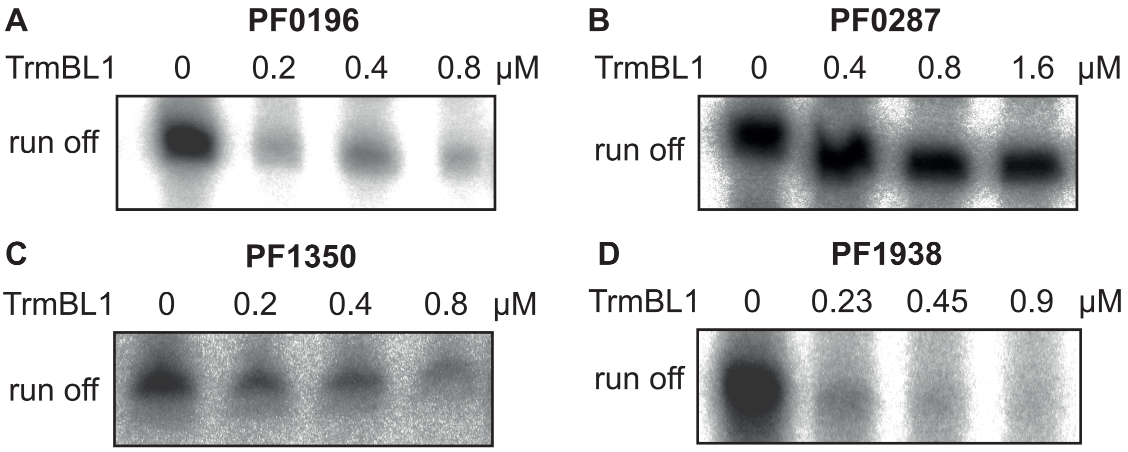

Additional file 8. *In vitro* transcription assays. *In vitro* transcription assays were done using as DNA templates promoters containing the TGM downstream or upstream of the BRE and TATA-box. Used TrmBL1 concentrations were indicated on top of each lane.
